# Parameter identifiability-based optimal observation remedy for biological networks

**DOI:** 10.1186/s12918-017-0432-2

**Published:** 2017-05-04

**Authors:** Yulin Wang, Hongyu Miao

**Affiliations:** 1 School of Computer Science and Engineering, University of Electronic Science and Technology of China, Chengdu, Sichuan China; 2 Department of Biostatistics, School of Public Health, University of Texas Health Science Center at Houston, Houston, TX 77030 USA

**Keywords:** Biological network, Graphical model, Structural identifiability analysis, Structural equation model, Observation strategy

## Abstract

**Background:**

To systematically understand the interactions between numerous biological components, a variety of biological networks on different levels and scales have been constructed and made available in public databases or knowledge repositories. Graphical models such as structural equation models have long been used to describe biological networks for various quantitative analysis tasks, especially key biological parameter estimation. However, limited by resources or technical capacities, partial observation is a common problem in experimental observations of biological networks, and it thus becomes an important problem how to select unobserved nodes for additional measurements such that all unknown model parameters become identifiable. To the best knowledge of our authors, a solution to this problem does not exist until this study.

**Results:**

The identifiability-based observation problem for biological networks is mathematically formulated for the first time based on linear recursive structural equation models, and then a dynamic programming strategy is developed to obtain the optimal observation strategies. The efficiency of the dynamic programming algorithm is achieved by avoiding both symbolic computation and matrix operations as used in other studies. We also provided necessary theoretical justifications to the proposed method. Finally, we verified the algorithm using synthetic network structures and illustrated the application of the proposed method in practice using a real biological network related to influenza A virus infection.

**Conclusions:**

The proposed approach is the first solution to the structural identifiability-based optimal observation remedy problem. It is applicable to an arbitrary directed acyclic biological network (recursive SEMs) without bidirectional edges, and it is a computerizable method. Observation remedy is an important issue in experiment design for biological networks, and we believe that this study provides a solid basis for dealing with more challenging design issues (e.g., feedback loops, dynamic or nonlinear networks) in the future. We implemented our method in R, which is freely accessible at https://github.com/Hongyu-Miao/SIOOR.

**Electronic supplementary material:**

The online version of this article (doi:10.1186/s12918-017-0432-2) contains supplementary material, which is available to authorized users.

## Background

The emergence of young research fields such as systems biology and network medicine [1, 2] reflects some exciting changes in biomedical investigators’ view of biology and practice. Particularly, it has been increasingly recognized that thinking in networks may lead to novel scientific insights and findings [3] that the traditional reductionism approaches cannot grant [4]. The recent development of experimental techniques (e.g., a variety of high-throughput omics approaches) also provides unprecedented opportunities for biomedical investigators to construct numerous biological networks at different levels and scales; for instance, protein-protein interaction networks [5, 6], gene regulatory networks [7–10], functional RNA networks [11–13], and metabolic networks [14, 15] can be found in a number of databases or knowledge repositories nowadays [9, 16, 17]. All such previous efforts provide a solid basis for further advancing our understanding of biological systems and the associated outcomes qualitatively or quantitatively.

Graphical models have long been considered as a natural mathematical representation of biological network for various quantitative analysis tasks such as parameter inference [18–21]. Specifically, given a biological network structure and experimental observations of certain variables associated with network nodes, it is often of significant interest to determine the unknown coefficients associated with network edges. For instance, to understand the responses of a biological network (e.g., activation or inhibition) to different environmental signals (e.g., different signaling molecules or different doses of the same signaling molecule), edge coefficients are likely to vary under different conditions and thus need to be estimated under each condition for the same given network structure [18]. In such a scenario, although the structure of the corresponding graphical model is known and fixed, concerns about the accuracy and reliability of parameter estimates often raise due to, e.g., the existence of unobserved node variables (i.e., latent variables). In practice, latent variables are not uncommon due to various technical limitations, ethic issues, financial affordability, and so on [18, 20]. Therefore, a natural question to ask is: what is the remedy that enables us to obtain reliable parameter estimates for a given graphical model structure with partially observed variables?

To the best knowledge of our authors, the aforementioned important question has rarely been tackled before in the context of quantifying unknown model parameters of biological networks; and in this study, we make the very first attempt to address this question from the structural identifiability point of view. By the definition in Miao et al. [18], an unknown model parameter is structurally identifiable if it can be uniquely determined for a given model structure under the assumptions that sample size is sufficiently large and data quality is not of concern. Of course, one can also take the effects of sample size and data noise into consideration and conduct the so-called practical identifiability analysis [18]; however, this is out of the scope of this study as practical identifiability analysis is not feasible at certain experimental design stage when real data are not available. On the contrary, structural identifiability analysis allows us to detect flaws in model structure and observation scheme before data collection, and thus should be investigated first. Our solution to the question mentioned at the end of the previous paragraph is thus a strategy that identifies a minimum number of unobserved nodes, for which the associated node variables should be observed in experiments such that all unknown parameters become structurally identifiable. This is a useful and cost-effective remedy if some of the model parameters are not identifiable given the original observation scheme, and we thus name it the structural identifiability-based optimal observation remedy (SIOOR).

Since biological networks can be represented by many different types of mathematical or statistical models, it is impossible to devise the SIOOR strategy for every different model type in one study. Therefore, we consider a linear structural equation model [22] here because it is a representative graphical model type and has been widely applied in various disciplines including systems biology [23–27]. A number of previous studies have investigated the parameter identifiability problem of SEMs, but the majority of these studies only derived theoretical criteria or conditions for identifiability verification, including Pearl’s back door and front door criteria [28], Brito and Pearl’s generalized instrumental variable criterion [29], Tian’s accessory set approach [30]. Only a few studies proposed computerizable identifiability analysis approaches, including Drton’s condition [31] and Foygel’s half-trek criterion [32] (implemented in R package SEMID), Sullivant’s computer algebra method and the more recent Wang’s identifiability matrix method [33, 34]. More importantly, all such criterions and methods assume that the observation strategy is given (i.e., it is pre-specified which variables are observed and which are not), and none of them considered the remedy strategy if a given observation strategy does not grant identifiability to all unknown model parameters. The focus of this study is thus to investigate how to choose a minimum number of nodes that are not observed in the original observation strategy for additional experimental measurements such that all unknown model parameters become identifiable. This study leads to a general and computerizable solution to the SIOOR problem for the first time.

More specifically, in the case that a given observation strategy of a biological network cannot grant identifiability of all unknown parameters in the corresponding SEM due to the existence of unobserved variables, we propose a dynamic programming (DP) approach to search for all possible SIOOR strategies. The proposed approach is a generic and computerizable method that can deal with recursive SEMs. It should be stressed that SIOOR strategy does not involve any power or sample size calculation and thus cannot be compared with the traditional experimental design approaches [35, 36]. Also, it should be stressed that the observability problem in control theory is different from the SIOOR problem because the aim of observability analysis is to determine the internal states of a system from its external outputs [37]. For clarification purpose, we also compare Liu’s graphic approach for observability analysis [38] with our SIOOR strategy in this study.

This article is organized as follows. In the Methods Section, the structural identifiability-based optimal observation remedy problem is mathematically formulated. We then propose a dynamic programming approach with theoretical justification to solve the problem for recursive SEMs. In the Results and Discussion Section, we describe our algorithm implementation and validate the proposed method using selected benchmark networks. Also, a real substructure from the influenza virus A [39] KEEG pathway is chosen as an example to illustrate the application of the proposed method in practice.

## Methods

In this section, several key concepts and definitions are introduced for solving the SIOOR problem, including Observation Strategy (OS), Cardinality of Observation Strategy [4], and Identifiability Gain (IG). The design of the dynamical programming algorithm is also described. In addition, we provide the necessary theoretical justification for the proposed method.

### Problem formulation

A directed biological network can be denoted by G = (**V, E**), where **V** denotes the node set and **E** denotes the edge set. Let *V*_*i*_ (*i* = 1, 2,…, *n*) denote the *i*-th node, and *Y*_*i*_ denote the variable associated with *V*_*i*_. If *Y*_*i*_ is a linear function of the remaining node variables, the corresponding SEM can be specified as follows,

$$ {Y}_i={\displaystyle \sum_{j\ne i}}{c}_{i j}{Y}_j+{\varepsilon}_i,\kern0.6em  i, j=1,\cdots, n, $$

where *c*_*ij*_ denotes the coefficient associated with the directed edge *V*_*j*_ → *V*_*i*_, and *ε*_*i*_ denotes the disturbance error term that follows a certain distribution (Gaussian or non-Gaussian [40, 41]) with mean zero. For simplicity, all disturbance error terms are assumed to be independent. By definition, **E** specifies the structure of the coefficient matrix **C** = [*c*_*ij*_], i.e., *c*_*ij*_ = 0 if no edge exists in **E** from *V*_*j*_ to *V*_*i*_ for *i* ≠ *j*. When a network structure contains one or more loops, G is a directed cyclic graph (DCG) and the corresponding SEM is called a non-recursive model; otherwise, G is a directed acyclic graph [42] and the corresponding SEM is called recursive. Although Drton’s condition [31] and Foygel’s half-trek criterion [32] are applicable to the identifiability analysis of non-recursive SEMs, the identifiability of parameters on a loop may be still inconclusive. Due to the lack of mature structural identifiability analysis techniques for examining every unknown parameter of a non-recursive SEM, this study focuses on recursive SEMs (i.e., DAGs) only.

**Definition 1** (*observation strategy*). Given a graph G = (**V**, **E**), its observation strategy can be denoted by a binary vector $O={\left({O}_{V_1},\cdots, {O}_{V_n}\right)}^T$, where ${O}_{V_i}=1$ if node *V*_*i*_ is observed and ${O}_{V_i}=0$ if *V*_*i*_ is unobserved. 

Observation strategy is important to parameter identifiability. In general, for a given network structure, the more observed nodes an observation strategy contains, the more likely all model parameters are identifiable. However, more observed nodes are usually associated with a higher experiment cost, so it is also desirable to reduce any unnecessary cost. The goal of SIOOR is thus to improve a given observation strategy by observing a minimum number of originally unobserved nodes such that all nonzero parameters in **C** become identifiable. For this purpose, let **P** denote the vector of all nonzero parameters in **C**, and let **D** denote the vector of identifiability status of every element in **P**. That is, if *P*_*i*_ is locally or globally identifiable (i.e., *P*_*i*_ has a finite number of possible values or a unique value within the parameter space, see [18]), *D*_*i*_ = 1; otherwise, *D*_*i*_ =1. When all the parameters in a model are locally or globally identifiable, this model is called identifiable. Consequently, the SIOOR problem can be formulated as follows

1 $$ \underset{\mathrm{observed}\ {V}_i}{ \min }{\displaystyle \sum_{i=1}^n{O}_{V_{{}_i}}},\ \mathrm{subject}\ \mathrm{t}\mathrm{o}\;\mathbf{D}=\mathbf{1}, $$

where ${\displaystyle \sum_{i=1}^n{O}_{V_{{}_i}}}$ is the total number of observed nodes in an observation strategy *O*, and **1** denotes a vector of ones. For clarification, we stress that the observation measurements are for the random variables associated with network nodes, and we assume (*n* – *m*) of them are observed in the original observation strategy, where *n* denotes the total number of nodes and 0 < *m* ≤ *n*.

The objective function above is minimized with respect to the originally unobserved nodes, subject to the constraint **D** = **1**. During the minimization process, it needs to be repeatedly verified whether all parameters have become identifiable (i.e., **D** = **1**). For this purpose, an efficient algorithm for structural identifiability analysis of SEMs is needed. Here we consider the identifiability matrix method proposed by Wang et al. [34]. Briefly, structural identifiability of parameters can be verified by examining the number of solutions to the symbolic polynomial identifiability equations generated by Wright’s path coefficient method [43, 44]. To avoid the expensive symbolic computation involved in reducing such identifiability equations, the identifiability matrix method proposes to derive binary matrices from symbolic polynomials and thus enable us to determine the number of solutions via several simple matrix operations. It is noteworthy that Wang’s work [34] does not explicitly handle colliders involving bidirectional arcs when generating identifiability equations with Wright’s method, however, the identifiability matrix method is still applicable here as we do not consider bidirectional arcs in DAGs.

### Identifiability gain and must-be-observed nodes

The optimization problem in the previous section is combinatorial in nature. Therefore, if the number of the originally unobserved nodes (denoted by *m*) is not small, enumerating all the 2^*m*^ different possible observation strategies over these nodes will be computationally expensive. We thus need an efficient algorithm such as dynamic programming to obtain the solutions. For this purpose, a few more definitions need to be introduced first.

**Definition 2** (*redundant identifiability equation*). Given a set of identifiability equations, an identifiability equation *IE*(*V*_*i*_,*V*_*j*_) is redundant with respect to that set if it can be expressed as a linear combination of the equations in that set.

**Definition 3** (*cardinality of observation strategy*). Given an observation strategy *O* for a network *G*, one symbolic polynomial identifiability equation can be generated for each pair of *d*-connected [28] observed nodes using, e.g., Wright’s path coefficient method. Then the total number of non-redundant identifiability equations is called the cardinality of *O*, denoted by *f*(*O*). 

The Wright’s path coefficient method generates identifiability equations for recursive SEMs by calculating the covariance between two node variables, which is equal to the sum of the products of edge coefficients along each *d*-connected path, i.e., $IE\left({V}_i,{V}_j\right):\  C o v\left({V}_i,{V}_j\right)={\displaystyle \sum_{pat{ h}_k}}{\displaystyle \prod_{e dg{ e}_l}}{\theta}_l$. After removing all redundant identifiability equations and redundant monomials, the identifiability result of each parameter can be determined by Theorem 1 in [34]. That is, if the number of non-redundant identifiability equations is less than the number of unknown parameters, then the parameters have an infinite number of possible values within the parameter space and are thus unidentifiable; otherwise, the parameters have a limited number of solutions or even a unique solution and are thus at least locally identifiable [45]. Let *N*_*u*_ denote the total number of unknown parameters in **P**. For every parameter in **P** being locally or globally identifiable, the inequality *f*(*O*) ≥ *N*_*u*_ should hold according to Theorem 1 in [34]. Therefore, the optimization problem can also be formulated as follows

2 $$ \underset{\mathrm{observed}\ {V}_i}{ \min }{\displaystyle \sum_{i=1}^n{O}_{V_{{}_i}}},\ \mathrm{subject}\ \mathrm{t}\mathrm{o}\; f(O)\ge {N}_u, $$

where the calculation of *f*(*O*) is a key challenge because it depends on specific network structure and observation strategy and thus has no closed-form solution. We thus introduce the following definition.

**Definition 4** (*identifiability gain*). Given a network G = (**V**,**E**), let *O* ^(*k*)^ and *f*(*O* ^(*k*)^) denote an observation strategy and its cardinality, respectively. Let *V*_*i*_ be an unobserved node in *O* ^(*k*)^, and only *V*_*i*_ becomes observed in a new observation strategy *O* ^(*k* + 1)^ with the observation statuses of other nodes remaining unchanged. Let *f*(*O* ^(*k* + 1)^) denote the cardinality of *O* ^(*k* + 1)^. Then the identifiability gain of observing *V*_*i*_, denoted by *g*(*V*_*i*_, *O* ^(*k*)^), is calculated as *g*(*V*_*i*_, *O* ^(*k*)^) = *f*(*O* ^(*k* + 1)^) − *f*(*O* ^(*k*)^). 

**Fig. 1:**
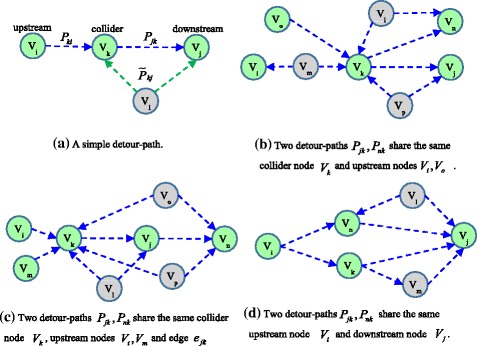
Four examples for illustrating the detour-path concept, where observed nodes are colored green and unobserved nodes are *colored grey*. **a** A simple detour-path; **b** Two detour-paths *P*_*jk*_, *P*_*nk*_ share the same collider node *V*_*k*_ and upstream nodes *V*_*i*_, *V*_*o*_; **c** Two detour-paths *P*_*jk*_, *P*_*nk*_ share the same collider node *V*_*k*_, upstream nodes *V*_*i*_, *V*_*m*_ and edge *e*_*jk*_; **d** Two detour-paths *P*_*jk*_, *P*_*nk*_ share the same upstream node *V*_*i*_ and downstream node *V*_*j*_

By definition, *g*(*V*_*i*_, *O* ^(*k*)^) is the difference in cardinality between two consecutive observation strategies *O* ^(*k*)^ and *O* ^(*k* + 1)^. That is, after *V*_*i*_ becomes observed in *O* ^(*k* + 1)^, we need to find out the number of newly added non-redundant identifiability equations. First, if another node *V*_*j*_ (*i* ≠ *j*) is observed in both *O* ^(*k*)^ and *O* ^(*k* + 1)^ and there exists a Wright’s path [46] of length 1 connecting *V*_*i*_ and *V*_*j*_, it can be shown that the newly added identifiability equation, denoted by *IE*(*V*_*i*_,*V*_*j*_), is non-redundant (see Lemma 1 and Additional file 1 for theoretical justification). However, if the length of every Wright’s path between *V*_*i*_ and *V*_*j*_ is greater than 1, the identifiability equation *IE*(*V*_*i*_,*V*_*j*_) is not always redundant, and it depends on both the node’s observation status and the structure of the network. Here we introduce the concept of detour-path before we further elucidate the redundancy issue. Consider a DAG G = (**V**,**E**) and two *d*-connected observed nodes *V*_*i*_ and *V*_*j*_. Assume that there exists a Wright’s path *P*_*ji*_ between *V*_*i*_ and *V*_*j*_ as well as an observed node *V*_*k*_(*k* ≠ *i*, *j*) on *P*_*ji*_, and the direction of *P*_*ji*_ is from *V*_*i*_ to *V*_*k*_ and then to *V*_*j*_. Now let *P*_*ki*_ and *P*_*jk*_ denote the two segments of *P*_*ji*_, then *P*_*ki*_ entering node *V*_*k*_ has an arrow pointing into *V*_*k*_ while *P*_*jk*_ exiting node *V*_*k*_ has an arrow pointing away from *V*_*k*_. However, if there exists another Wright’s path between *V*_*k*_ and *V*_*j*_, denoted by ${\tilde{P}}_{kj}$, which has no any other observed nodes besides *V*_*k*_ and *V*_*j*_ and has an arrow pointing into *V*_*k*_, then *V*_*k*_ is a collider with respect to *P*_*ki*_ and ${\tilde{P}}_{kj}$. Thus, we call the Wright’s path segment *P*_*jk*_ the detour-path, and call *V*_*i*_, *V*_*j*_ and *V*_*k*_ the upstream node, the downstream node, and the collider node of the detour-path *P*_*jk*_, respectively. By definition, a detour-path can have only one downstream node and one collider node but may have one or more upstream nodes. Moreover, multiple detour-paths can share the same upstream node, the same downstream node or the same collider node. Several examples are given in Fig. 1 to illustrate the concept of detour-path.

In addition, when an upstream node *V*_*i*_ is shared by two or more detour-paths that have the same downstream node, *V*_*i*_ is called a shared upstream node; otherwise, *V*_*i*_ is called an exclusive upstream node. Note that a detour-path can have both exclusive and shared upstream nodes in the same time, and the collider node of one detour-path can be an upstream node of another detour-path. Consider two detour-paths that have no exclusive upstream nodes, if they share the same downstream node and at least one upstream node, or one upstream node of one detour path is the collider node of the other detour-path, then two detour-paths are intersecting. One can tell that if ${P}_{j{ k}_1}$ intersects with ${P}_{j{ k}_2}$ and ${P}_{j{ k}_2}$ intersects with ${P}_{j{ k}_3}$, then ${P}_{j{ k}_1}$ also intersects with ${P}_{j{ k}_3}$. Then we consider a downstream node *V*_*j*_, let *S*_*IDP* denote all the intersecting detour-paths, and let *S*_*SUN* denote all the shared upstream nodes of *S*_*IDP*. Similar to a single unknown parameter, the coefficient product $W P={\displaystyle \prod_{e dg{ e}_l}}{\theta}_l$ of a Wright’s path *P* can be deemed as a single parameter and one can tell its structural identifiability based on identifiability equations. If a detour-path *P* has at least one exclusive upstream node, then the Wright’s coefficient *WP* of *P* is globally identifiable (see Lemma 2 and Additional file 1 for theoretical justification). Also, for a group of intersecting detour-paths, if the node number of *S*_*SUN* is equal to or greater than the number of intersecting detour-paths in *S*_*IDP*, then the Wright’s coefficient of each detour-path in *S*_*IDP* is globally identifiable (see Lemma 3 and Additional file 1 for theoretical justification).

Given a DAG G = (**V**,**E**), consider two observed nodes *V*_*i*_, *V*_*j*_ and an unobserved node *V*_*u*_. *V*_*u*_ may not be on any Wright’s paths between *V*_*i*_ and *V*_*j*_. For this case,if only *V*_*u*_ becomes observed in *O* ^(*k* + 1)^, then for each observed node *V*_*i*_ in *O* ^(*k*)^, one can check whether the identifiability equation *IE*(*V*_*i*_,*V*_*u*_) is redundant according to Lemma 4 (see Additional file 1 for theoretical justification). That is, when none of the Wright’s paths between *V*_*i*_ and *V*_*u*_ contains detour-paths, *IE*(*V*_*i*_,*V*_*u*_) is redundant if and only if each Wright’s path between *V*_*i*_ and *V*_*u*_ passes at least one observed node other than *V*_*i*_ and *V*_*u*_; otherwise, *IE*(*V*_*i*_,*V*_*u*_) is redundant if and only if the Wright’s coefficient of each detour-path between *V*_*i*_ and *V*_*u*_ is globally identifiable in *O* ^(*k*)^ and each Wright’s path between *V*_*i*_ and *V*_*u*_ passes at least one observed node other than *V*_*i*_ and *V*_*u*_. If *V*_*u*_ is on a Wright’s path between *V*_*i*_ and *V*_*j*_, and the sufficient and necessary condition for one of the identifiability equations *IE*(*V*_*i*_,*V*_*u*_) and *IE*(*V*_*j*_,*V*_*u*_) being redundant is similar to Lemma 4 and given in Lemma 5 (see Additional file 1 for theoretical justification). Note that it can be determined whether the Wright’s coefficient of a detour-path is globally identifiable according to Lemma 2 and Lemma 3.

Based on Lemma 4 and Lemma 5, we propose a novel graphic method to calculate the identifiability gain *g*(*V*_*i*_, *O* ^(*k*)^). Let *des*_*i*_ denote the descendant node set of *V*_*i*_, *anc*_*i*_ denote the ancestor node set of *V*_*i*_, *rel*_*i*_ denote the set of nodes that are not included in *des*_*i*_ or *anc*_*i*_. Moreover, let *bound*_*i*_ ⊂ *anc*_*i*_ denote the boundary node set, in which every node has at least one outgoing edge to a node in *rel*_*i*_. Then we can calculate *g*(*V*_*i*_, *O* ^(*k*)^) by removing the following edges from the original graph **G**: i) all the incoming edges to the observed nodes that are not collider nodes of detour-paths in *anc*_*i*_; ii) all the outgoing edges from some observed nodes in *des*_*i*_ and *rel*_*i*_ and these observed nodes are not the collider nodes of the detour-paths whose Wright’s coefficients are unidentifiable in *O* ^(*k*)^; and iii) all the outgoing edges from the observed nodes in *bound*_*i*_ to nodes in *rel*_*i*_, and then we get a new graph denoted by G '. Let *N*_*w*_ denote the total number of the observed nodes that are connected with *V*_*i*_ via any Wright’s path in graph G '. Furthermore, one can tell from the edge-removal operation that there still exist some redundant identifiability equations in G ', because the following two types of redundancy cases have not been considered in the edge-removal operation: *V*_*i*_ is the downstream node of an arbitrary detour-path, and *V*_*i*_ is on a Wright’s path between two observed nodes in G '. Let *N*_*r*_ denote the number of redundant identifiability equations in G '. According to the topological structure of G ' and the node’s observation status, we can obtain *N*_*r*_ based on Lemma 4 and Lemma 5 (see the details in Implementation and Verification Section). It can be shown that the identifiability gain is *g*(*V*_*i*_, *O* ^(*k*)^) = *N*_*w*_ − *N*_*r*_ (see Theorem 1 and Additional file 1 for theoretical justification).

For a given DAG **G** and an observation strategy *O* ^(*k*)^, different unobserved nodes may associate with different identifiability gains. Naturally, our strategy is to choose the unobserved node in *O* ^(*k*)^ with the maximum identifiability gain if it becomes observed in *O* ^(*k* + 1)^. However, we also recognize that, to assure that all model parameters are at least locally identifiable, certain nodes of a DAG must be observed if they are unobserved in an observation strategy (see Lemma 6 and Additional file 1 for theoretical justification). For convenience, we call such nodes the must-be-observed [14] nodes, and let ${O}^{(0)_M}$ denote the observation strategy, in which only the MBO nodes are observed.

**Lemma 1** *Given a DAG* G = (***V***,***E***), *an observed node* *V*_*i*_, *and an unobserved node* *V*_*u*_ *in* *O* ^(*k*)^, *if only* *V*_*u*_ *becomes observed in* *O* ^(*k* + 1)^, *the identifiability equation* *IE*(*V*_*i*_,*V*_*u*_) *is non-redundant if there exists a Wright’s path of length 1 connecting* *V*_*i*_ *and* *V*_*u*_.

**Lemma 2** *If a detour-path* *P* *has one or more exclusive upstream node, the Wright’s coefficient* *WP* of *P* *is globally identifiable*.

**Lemma 3** *For a group of intersecting detour-paths, if the number of the shared upstream nodes in* *S*_*SUN* *is equal to or greater than the number of intersecting detour-paths in* *S*_*IDP*, *then the Wright’s coefficient of each detour-path in* *S*_*IDP* *is globally identifiable*.

**Lemma 4** *Given a DAG* G = (**V**,**E**), *an observed node* *V*_*i*_, *and an unobserved node* *V*_*u*_ in *O* ^(*k*)^, *if only* *V*_*u*_ *becomes observed in* *O* ^(*k* + 1)^, *there exist two cases:*

1. each Wright’s path between *V*_*i*_ and *V*_*u*_ passes at least one observed node other than *V*_*i*_ and *V*_*u*_ when none of the Wright’s paths between *V*_*i*_ and *V*_*u*_ contains detour-paths;
2. each Wright’s path between *V*_*i*_ and *V*_*u*_ passes at least one observed node other than *V*_*i*_ and*V*_*u*_, and the Wright’s coefficient of each detour-path between *V*_*i*_ and *V*_*u*_ is globally identifiable in *O* ^(*k*)^ when certain Wright’s paths between *V*_*i*_ and *V*_*u*_ contain detour-paths.

Then the identifiability equation *IE*(*V*_*i*_,*V*_*u*_) is redundant if and only if one of the above conditions holds.

**Lemma 5** *Given a DAG* G = (**V**,**E**), *two* *d*-*connected observed nodes* *V*_*i*_ *and* *V*_*j*_, *and an unobserved node* *V*_*u*_ *in O* ^(*k*)^, *if V*_*u*_ *is on a Wright’s path between V*_*i*_ *and V*_*j*_ *and only V*_*u*_ *becomes observed in O* ^(*k* + 1)^, *there exist two cases:*

1. each Wright’s path between *V*_*i*_ and *V*_*j*_ passes at least one observed node other than *V*_*i*_ and *V*_*j*_ when none of the Wright’s paths between *V*_*i*_ and *V*_*j*_ contains detour-paths;
2. each Wright’s path between *V*_*i*_ and *V*_*j*_ passes at least one observed node other than *V*_*i*_ and *V*_*j*_, and the Wright’s coefficient of each detour-path between *V*_*i*_ and *V*_*j*_ is globally identifiable in *O* ^(*k*)^ when certain Wright’s paths between *V*_*i*_ and *V*_*j*_ contain detour-paths.

Then one of the two identifiability equations *IE*(*V*_*i*_,*V*_*u*_) and *IE*(*V*_*j*_,*V*_*u*_) is redundant if and only if one of the above conditions holds.

**Theorem 1** *Given a DAG* G = (**V**, **E**) *and an unobserved node V*_*i*_ *in an observation strategy O*, *let* G ' *denote the sub-graph after the edge-removal operation. Then the identifiability gain is g*(*V*_*i*_, *O*) = *N*_*w*_ − *N*_*r*_, *where N*_*w*_ *denotes the total number of the observed nodes that are connected with V*_*i*_ via *any Wright’s path in graph* G ', *and N*_*r*_ *denotes the number of redundant identifiability equations in graph* G '.

**Lemma 6** *For a given DAG* G = (**V**, **E**), *the following nodes must be observed to assure that all the parameters of the corresponding SEM are at least locally identifiable*

1. The nodes with an out-degree 0;
2. The nodes with an out-degree 1;
3. The nodes with an in-degree 0 and an out-degree less than 3.

### Dynamic programming strategy

Let ${O}^{(0)_G}$ denote a given observation strategy. If some of the MBO nodes are not observed in ${O}^{(0)_G}$, ${O}^{(0)_M}$ should be incorporated into ${O}^{(0)_G}$ according to Lemma 6. Therefore, the initial observation strategy, denoted by *O* ^(0)^, should always be ${O}^{(0)}=\left({O}^{(0)_M}\Big|{O}^{(0)_G}\right)$, where the OR operator is an element-wise operation. For example, for a DAG with six nodes, if $$ {O}^{(0)_M}={\left[\begin{array}{cccccc}\hfill 1\hfill & \hfill 0\hfill & \hfill 1\hfill & \hfill 0\hfill & \hfill 0\hfill & \hfill 0\hfill \end{array}\right]}^T $$ and $$ {O}^{(0)_G}={\left[\begin{array}{cccccc}\hfill 0\hfill & \hfill 1\hfill & \hfill 1\hfill & \hfill 0\hfill & \hfill 0\hfill & \hfill 0\hfill \end{array}\right]}^T $$, then $$ {O}^{(0)}={\left[\begin{array}{cccccc}\hfill 1\hfill & \hfill 1\hfill & \hfill 1\hfill & \hfill 0\hfill & \hfill 0\hfill & \hfill 0\hfill \end{array}\right]}^T $$.

The dynamic programming strategy starts with the calculation of the cardinality of *O* ^(0)^ (that is, *f*(*O* ^(0)^)) based on Theorem 1. Specifically, let *R* be the number of observed nodes in *O* ^(0)^, *V*_*o*_*r*_(*r* = 1, 2, ⋯, *R*) be the *r*-th observed node in *O* ^(0)^, and *O* ^(0)^{*V*_*o*_1_, …, *V*_*o*_*r*_} be the observation strategy in which only the first *r* observed nodes in *O* ^(0)^ are observed. Then $f\left({O}^{(0)}\right)={\displaystyle \sum_{r=1}^{R-1} g\left({V}_{o\_\left( r+1\right)},{O}^{(0)}\left\{{V}_{o\_1},\dots, {V}_{o\_ r}\right\}\right)}$ can be calculated according to Theorem 1. Note that the order at which *V*_*o*_*r*_ is selected into *O* ^(0)^{*V*_*o*_1_, …, *V*_*o*_*R*_} will not change the observation strategy (e.g., *O* ^(0)^{*V*_*o*_1_, *V*_*o*_2_} = *O* ^(0)^{*V*_*o*_2_, *V*_*o*_1_}) and thus have no effect on the value of *f*(*O* ^(0)^).

**Fig. 2:**
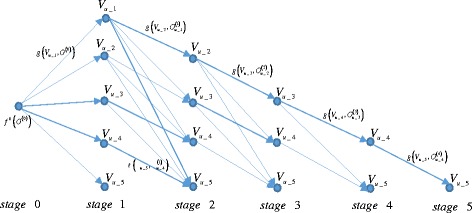
Schematic illustration of the stages, states and state transition costs in the proposed dynamic programming strategy

The second step of our dynamic programming strategy is to define stages and their associated states. Let *S* denote the number of unobserved nodes in *O* ^(0)^, and let *V*_*u*_*s*_ (*s* = 1, 2, ⋯, *S*) denote the *s*-th unobserved node in *O* ^(0)^, then the dynamic programming procedure can be divided into *S* + 1 stages. For illustration purpose, we consider a simple example with 5 unobserved nodes, as shown in Fig. 2. The 0-th stage is actually the initialization step as described in the previous paragraph, and it has only one state, i.e., *O* ^(0)^. At the first stage, there are *S* = 5 different states; that is, only one of the unobserved nodes {*V*_*u*_1_, *V*_*u*_2_, ⋯, *V*_*u*_5_} in *O* ^(0)^ will be selected to observe. At the second stage, since one of the five unobserved nodes has been selected at the previous stage, there are only four unobserved nodes for selection and thus four states exist (that is, {*V*_*u*_2_, *V*_*u*_3_, *V*_*u*_4_, *V*_*u*_5_}). Therefore, as shown in Fig. 2, except for stages 0 and 1, each subsequent stage has one less states than its previous stage; also, the upper triangular region (see the area above the labels of stages 1–5 in Fig. 2) is empty because the selection order of unobserved nodes does not affect the eventual observation strategy so the inclusion of such states in the upper triangular region is redundant. One can tell that the proposed stage and state definitions satisfy the optimality principle of dynamic programming [47–49].

The third step is to compute the state transition costs for searching the optimal state transition path(s). According to the definitions of stages and states, there may exist several different states at the *s*-th stage that can transit to the same state at the (*s* + 1)-th stage. For instance, four states *V*_*u*_1_, *V*_*u*_2_, *V*_*u*_3_ and *V*_*u*_4_ at the first stage can transit to *V*_*u*_5_ at the second stage, as shown in Fig. 2. The state transition cost from state *V*_*u*_*i*_ to state *V*_*u*_*j*_ (*i* ≠ *j*) between two consecutive stages is just the identifiability gain *g*(*V*_*u*_*j*_, *O* ^(*k*)^{…, *V*_*u*_*i*_, …}), where *O* ^(*k*)^{…, *V*_*u*_*i*_, …} means that *V*_*u*_*i*_ is observed in *O* ^(*k*)^. Then the cardinality of an observation strategy can be computed by adding *f*(*O* ^(0)^) and all the state transition costs along the state transition path. Since the goal of the dynamic programming strategy is to search for the optimal observation strategies, when there exist multiple transition paths from state *V*_*u*_*i*_ in *O* ^(*k*)^ to state *V*_*u*_*j*_ in *O* ^(*k* + 1)^ (*i* ≠ *j*), the transition path associated with the maximum identifiability gain will be chosen; that is, $f\left({O}_{V_{u\_ j}}^{\left( k+1\right)}\right)=\underset{V_{u\_ i}, i\ne j}{ \max}\left( g\left({V}_{u\_ j},{O}_{V_u{\_}_i}^{(k)}\right)+ f\left({O}_{V_{u\_ i}}^{(k)}\right)\right)$, where ${O}_{V_{u\_ i}}^{(k)}$ is a convenient notation for *O* ^(*k*)^{…, *V*_*u*_*i*_, …}.

The dynamic programming strategy above can be mathematically described in Eq. (3), and we have implemented this strategy in R (see the “Implementation and verification” Section),

3 $$ \left\{\begin{array}{l} f\left({O}_{V_{u\_ s}}^{(1)}\right)= f\left({O}^{(0)}\right)+ g\left({V}_{u\_ s},{O}^{(0)}\right),\kern0.75em  s=1,2,\dots, S,\\ {} f\left({O}_{V_{u\_ j}}^{\left( k+1\right)}\right)=\underset{V_{u\_ i}, i\ne j}{ \max}\left( g\left({V}_{u\_ j},{O}_{V_{u\_ i}}^{(k)}\right)+ f\left({O}_{V_{u\_ i}}^{(k)}\right)\right),\kern0.75em  k=1,2,\cdots\ \&\  k\le S-1.\end{array}\right. $$

It should be stressed that it is not necessary to finish all the *S* iterations as shown in Eq. (3). Once the cardinality $f\left({O}_{V_{u\_ i}}^{(k)}\right)$ at the *k*-th stage becomes equal to or greater than the number of unknown parameters *N*_*u*_, the dynamic programming process will stop and we get the SIOOR strategies.

## Results and Discussion

### Overview of the framework

Observation strategy design is an under-investigated problem for biological networks, despite the fact that a variety of biological networks have been actively constructed and used in numerous benchside or bedside studies. However, the existence of latent variables is a common problem due to cost, technical or other limitations, and has significantly hampered our capability to quantitatively investigate and understand such networks via, e.g., key network parameter estimation from experimental data. Identifiability analysis has long been recognized as a powerful tool to assure the accuracy and reliability of parameter estimation techniques; however, identifiability-based observation strategy design for biological networks turns out to be an unexplored field despite its substantial importance to biological network studies like structure identification.

To the best knowledge of our authors, this is the first study that tackles the problem of identifiability-based observation strategy design for biological networks described by linear SEMs. First, we introduce several new concepts such as cardinality of observation strategy and identifiability gain and mathematically formulate the identifiability-based optimal observation problem. Second, for a given network structure, the key idea is to turn a minimum number of unobserved nodes in the original observation strategy into observed such that the number of non-redundant identifiability equations becomes greater than or equal to the number of unknown model parameters (i.e., the whole system is at least locally identifiable). By counting the number of non-redundant identifiability equations, we avoid performing actual identifiability analysis on SEM and the proposed method is thus computationally efficient. Third, by defining the concepts of stage division and state transition, a dynamic programming strategy is proposed to solve the maximization problem without involving any time-consuming symbolic computation or matrix operations [33, 34]. Fourth, an efficient computing algorithm is proposed to calculate the identifiability gain of each unobserved node in a given observation strategy. More specifically, the computing process is significantly simplified by counting the number of observed nodes that connect with the node of concern via Wright’s paths after removing certain edges from the original graph.

It takes a non-constant time to compute the node identifiability gain in each iteration, and the algorithm complexity depends on the number of observed nodes. Furthermore, the number of iterations of the dynamic programming algorithm does not depend on the total number of nodes, but the number of unobserved nodes in the original observation strategy. Let *S* denote the number of unobserved nodes and *T* denote the number of observed nodes in the original observation strategy, then the computation complexity of the dynamic programing strategy is *O*(*S* ^2^ ⋅ *T*).

### Implementation and verification

**Fig. 3:**
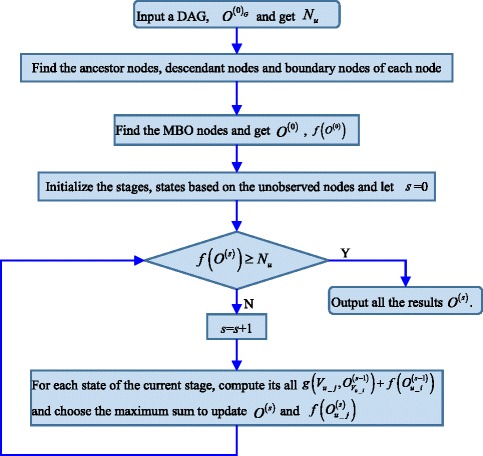
Flowchart of the proposed dynamic programming algorithm

The flowchart of the proposed algorithm for searching the structural identifiability-based optimal observation remedy is shown in Fig. 3. We have implemented the dynamic programming algorithm in R, and all the source codes and examples are freely accessible at https://github.com/Hongyu-Miao/SIOOR.

Here we describe several important technical details of the implementation. First, at the state transition step, i.e., $f\left({O}_{V_{u\_ j}}^{\left( k+1\right)}\right)=\underset{V_{u\_ i}, i\ne j}{ \max}\left( g\left({V}_{u\_ j},{O}_{V_{u\_ i}}^{(k)}\right)+ f\left({O}_{V_{u\_ i}}^{(k)}\right)\right)$, if there exist multiple transitions that produce the same *f*(*O* ^(*k* + 1)^), our current implementation chooses only one such transition to update the next-stage observation strategy. If needed, the R code can be slightly modified to enumerate all optimal observation strategies. Second, since the boundary node set is just a subset of the ancestor node set for a given node, the processing of the boundary nodes is incorporated into the processing of the ancestor nodes in the current implementation.

**Fig. 4:**
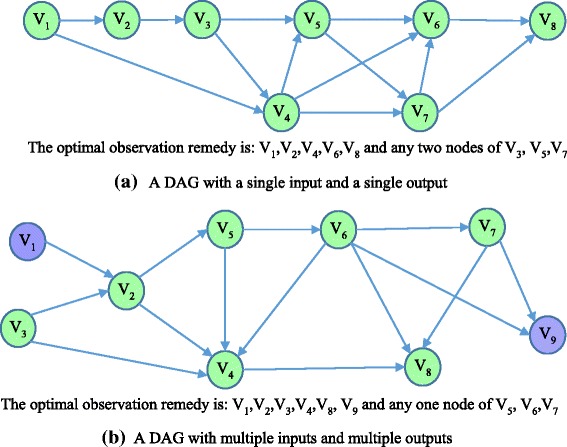
Two DAG examples for algorithm implementation validation, where the *green nodes* are unobserved and the *blue* ones are observed in the initial observation strategy. **a** A DAG with a single input and a single output; **b** A DAG with multiple inputs and multiple outputs

In order to verify the implementation, synthetic DAGs can be generated for this purpose, like the two DAG examples in Fig. 4. The first DAG contains 8 nodes and 13 edges, and it has only a single input node and a single output node. Moreover, the first example considers a special initial observation strategy (i.e., all nodes are unobserved) to illustrate the capability of the proposed method to design optimal observation strategy from scratch. The second DAG has multiple input and output nodes, and it considers a more general situation, that is, there exists both observed and unobserved nodes in the initial observation strategy. We analyzed the two examples using the proposed algorithm, and used the identifiability matrix method [34] to verify that the obtained observation remedies do grant (local) identifiability to all model parameters.

### Applications to real biological networks

Since it is impossible to cover all the biological networks in various databases and knowledge repositories [16, 17] in one study, we choose the biological network associated with influenza A virus [39] as an application example for illustration purpose. IAV can infect birds as well as mammals including human, and it has been one of the major infectious pathogens that have caused millions of human deaths. It is thus of great scientific significance to systematically understand IAV infection and immune response mechanisms. Therefore, Matsuoka et al. [50] manually curated a comprehensive database, called FluMap, for depicting the influenza virus life cycle at the molecular level from over 500 previous publications. There are mainly five modules in FluMap: virus entry, virus replication and transcription, post-translational processing, transportation of virus proteins, and packaging and budding. Given the critical role of virus replication in influenza virus life cycle, numerous experimental studies (e.g., [42, 51–53]) have made attempts to understand virus replication mechanisms and their clinical implications. Thus, we choose to focus on the IAV replication module and analyze its observation strategy.

**Fig. 5:**
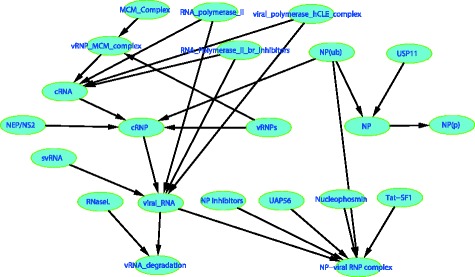
An application example based on the influenza A virus replication module, where all nodes are initially unobserved and in *green color*

Since IAV replication involves many different biomolecules and complex interactions, it is usually infeasible to observe all such components and their interactions in one study. The question of concern here is how to choose a minimal number of nodes in Fig. 5 to observe such that all the model parameters become at least locally identifiable. Note that Fig. 5 is derived from Matsuoka’s work [50], and consists of 22 nodes and 26 edges; for simplicity, the catalyzers and inhibitors in this network are treated as reactants.

**Fig. 6:**
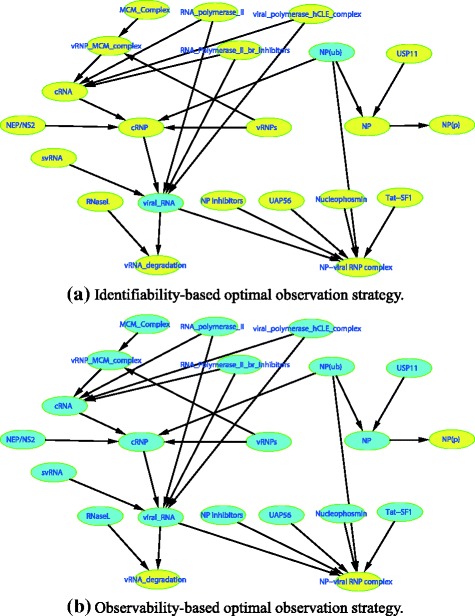
The optimal observation strategies for the influenza A virus replication module based on **a** identifiability and **b** observability, where the *yellow nodes* are observed and the *green nodes* are unobserved

A relevant concept, called observability, has been previously investigated by Liu et al. [38] for complex dynamic systems. Although observability analysis also deals with observation strategies considering the existence of latent variables, it is very different from identifiability analysis in two aspects: 1) the focus of observability analysis is not model parameters but how to infer the unobserved state variables from experimentally measured outputs of a system; 2) the graphical approach proposed by Liu et al. was developed for the so-called balance equations based on mass-action kinetics, the model structures of which are very different from static linear SEMs. However, it is of interest to compare the identifiability-based observation results with those of the observability-based method. For this purpose, we assume that all the nodes in Fig. 5 are initially unobserved. After applying the proposed dynamic programing method, we get the optimal observation strategy shown in Fig. 6(a) for achieving parameter identifiability. The optimal observation strategy produced by Liu’s observability approach is shown in Fig. 6(b). According to Fig. 6(a) and (b), one can tell that the identifiability-based observation strategy contains 20 observed nodes and 2 unobserved nodes, while the observability-based strategy contains 3 observed nodes and 19 unobserved nodes. That is, for the IAV replication module, the system internal states can be inferred from a few observed output nodes if a balance equation model is used; however, it needs much more observed nodes to achieve parameter identifiability if a linear SEM is used. Such an observation is not only due to the different goals of observability and identifiability analyses, but also the differences in the underlying model structures used in observability or identifiability analyses.

Moreover, besides the nodes with an out-degree 0 or 1 as mentioned in Lemma 3, the identifiability-based observation strategy is also likely to select the nodes with a high out-degree as unobserved nodes; for instance, the two unobserved nodes viral_RNA and NP(ub) in Fig. 6(a) have the highest out-degrees 2 and 3, respectively. This is because, if an unobserved node has a high out-degree, this node is connected with many out-neighbor nodes; when its out-neighbor nodes are observed, there will exist multiple Wright’s paths that connect such out-neighbor nodes and pass this unobserved node, and the corresponding identifiability equations thus contain the parameters associated with the out-edges of this unobserved node such that these parameters can be identifiable. Interestingly, the observability-based strategy tends to select the nodes with a low out-degree as observed nodes, for example, all the nodes with 0 out-degree are observed in Fig. 6(b). It is because the nodes with an out-degree 0 in a DAG are usually the final products of chemical reactions, instead of reactants, and thus the internal states associated with other nodes can be easily inferred based on the balance equations if all the final products of chemical reactions are measured.

## Conclusions

In this study, we address an important problem for biological networks: the design of observation strategies for all edge coefficients being identifiable. Linear SEMs are used as the mathematical representation of biological networks, which allows us to formulate the problem as a constrained optimization problem. A dynamic programming strategy was then developed to solve the constrained optimization problem to obtain the optimal observation strategies at the cost of turning a minimal number of unobserved nodes into observed. The proposed solution is novel and efficient because it avoids both symbolic computation and matrix operations as used in other studies, and we provided necessary theoretical justifications for the proposed algorithm. As verified by multiple examples (synthetic or real networks), the proposed solution is generic and can be applied to an arbitrary DAG (recursive SEMs) without bidirectional edges.

We also recognize that many real biological networks are dynamic, nonlinear, or have feedback loops, which are beyond the capability of the method developed in this study. However, this study provides a basis for determining the identifiability-based optimal observation remedy for more complex biological networks, and we expect to tackle the more challenging problems in the future.

## Additional file

Additional file 1: Theoretical justifications for identifiability gain computation. (PDF 217 kb)

## Abbreviations

SEM: Structure equation model
DAG: Directed acyclic graph
SIOOR: Structural identifiability-based optimal observation remedy
IAV: Influenza A virus
DP: Dynamic programming
OS: Observation strategy
IG: Identifiability gain
DCG: Directed cyclic graph
