# Supplementary material for: Parameter identifiability-based optimal observation remedy for biological networks
Source: BMC Syst Biol. 2017 May 4;11:53. doi: 10.1186/s12918-017-0432-2 (PMC5418771; doi:10.1186/s12918-017-0432-2)
Supplement: Additional file 1: — Theoretical justifications for identifiability gain computation. (PDF 217 kb) [file 12918_2017_432_MOESM1_ESM.pdf]

Supplementary Materials

Theoretical justification for identifiability gain computation

Yulin Wang, Hongyu Miao

Lemma 1. Given a DAG $\mathrm{G}=(\mathbf{V},\mathbf{E})$ and an observed node V_i and an unobserved node V_u in $O^{(k)}$, if only V_u becomes observed in $O^{(k+1)}$, then the identifiability equation $IE(V_i,V_u)$ is non-redundant if there exists a Wright's path of length 1 connecting V_i and V_u.

Proof. Without loss of generality, assume that there exists $k+1$ $(k \geq 0)$ Wright's paths connecting V_i and V_u. The path of length 1 is just the edge directly from V_i to V_u associated with coefficient c_{ui}, and the lengths of the remaining k Wright's paths are assumed greater than 1. Let P_{k+1} denote the Wright's path of length 1, $P_l\,(l=1,2,\cdots,k)$ the rest of the Wright's paths, and WP the Wright's path coefficient of a path, then the newly-added identifiability equation is $IE(V_i,V_u)$: $Cov(V_i,V_u)=\sum_{l=1}^{k} WP_l + WP_{k+1}$ according to Wright's path coefficient method [1, 2]. WP_{k+1} can be simply replaced with c_{ui} to obtain $IE(V_i,V_u)$: $Cov(V_i,V_u)=\sum_{l=1}^{k} WP_l + c_{ui}$. Since V_j is unobserved in $O^{(k)}$, all the identifiability equations generated from $O^{(k)}$ do not contain a term that consists of only parameter c_{ui}. Therefore, $IE(V_i,V_u)$ cannot be expressed as a linear combination of all the identifiability equations from $O^{(k)}$. That is, $IE(V_i,V_u)$ is non-redundant if there exists a Wright's path of length 1 connecting V_i and V_u. ■

Lemma 2. If a detour-path P has one or more exclusive upstream node, the Wright's coefficient WP of P is globally identifiable.

Proof. Without loss of generality, let V_i, V_j and V_k denote an exclusive upstream node, the downstream node, and the collider node of P, respectively. Let S_WP_{ki} denote the coefficient sum of Wright's paths between V_i and V_k, S_DWP_{jk} the Wright's path coefficient of P, and S_CWP_{jk} the Wright's path coefficient sum of all the Wright's paths between V_j and V_k that collide with the path from V_i to V_k. Since V_i, V_j and V_k are observed nodes, one can get the following three identifiability equations:

$$IE\left(V_i, V_k\right): \ Cov\left(V_i, V_k\right) = S\_WP_{ki},$$

$$IE\left(V_i, V_j\right): \ Cov\left(V_i, V_j\right) = S\_WP_{ki} \cdot S\_DWP_{jk},$$

$$IE\left(V_j, V_k\right): \ Cov\left(V_j, V_k\right) = S\_DWP_{jk} + S\_CWP_{jk}.$$

One can tell from the above three equations that S_WP_{ki}, S_DWP_{jk} and S_CWP_{jk} have a unique solution and are thus globally identifiable. Therefore, the Wright's coefficient WP of P is globally identifiable.

Lemma 3. For a group of intersecting detour-paths, if the number of the shared upstream nodes in S_SUN is equal to or greater than the number of intersecting detour-paths in S_IDP, then the Wright's coefficient of each detour-path in S_IDP is globally identifiable.

Proof. Without loss of generality, assume that there are $n\left(n \geq m\right)$ shared upstream nodes $V_{i_1}, V_{i_2}, \cdots, V_{i_n}$ in S_SUN and there are m detour-paths $P_1, P_2, \cdots, P_m$ in S_IDP. Let $V_{k_1}, V_{k_2}, \cdots, V_{k_m}$ denote the m collider nodes respectively, and V_j denote

the downstream node of $P_1, P_2, \cdots, P_m$. Consider an upstream node V_{i_p} and a collider node V_{k_q}, and there exists a path from V_{i_p} to V_{k_q}. We can get an identifiability equation $IE\left(V_{i_p}, V_{k_q}\right)$: $Cov\left(V_{i_p}, V_{k_q}\right) = S\_WP_{i_p k_q}$. One can tell from $IE\left(V_{i_p}, V_{k_q}\right)$ that $S\_WP_{i_p k_q}$ is globally identifiable.

Similarly, one can get that all the Wright's path coefficients $S\_WP_{i_p k_q}$ between $V_{i_1}, V_{i_2}, \cdots, V_{i_n}$ and $V_{k_1}, V_{k_2}, \cdots, V_{k_m}$ are globally identifiable. Then we consider an upstream node V_{i_p} and two collider nodes V_{k_q}, V_{k_r}, where V_{i_p} has directed paths to V_{k_q} and V_{k_r}, respectively. We can get an identifiability equation $IE\left(V_{k_q}, V_{k_r}\right)$: $Cov\left(V_{k_q}, V_{k_r}\right) = S\_WP_{i_p k_q} \cdot S\_WP_{i_p k_r}$. Because $S\_WP_{i_p k_q}$ and $S\_WP_{i_p k_r}$ are globally identifiable, one can tell that the identifiability equation $IE\left(V_{k_q}, V_{k_r}\right)$ is redundant. Similarly, one can tell that all the identifiability equations between $V_{k_1}, V_{k_2}, \cdots, V_{k_m}$ are redundant.

Finally, we consider the identifiability equations between $V_{i_1}, V_{i_2}, \cdots, V_{i_n}$ and V_j. We can get n identifiability equations between $V_{i_1}, V_{i_2}, \cdots, V_{i_n}$ and V_j. These equations contain all the Wright's path coefficients from $V_{i_1}, V_{i_2}, \cdots, V_{i_n}$ to $V_{k_1}, V_{k_2}, \cdots, V_{k_m}$, and m Wright's path coefficients from each node of $V_{k_1}, V_{k_2}, \cdots, V_{k_m}$ to V_j. Since each Wright's path coefficient $S\_WP_{i_p k_q}$ from $V_{i_1}, V_{i_2}, \cdots, V_{i_n}$ to $V_{k_1}, V_{k_2}, \cdots, V_{k_m}$ is globally identifiable, the n identifiability equations contains only m unknown Wright's path coefficients. One can tell from $n \geq m$ that each Wright's path coefficient from $V_{k_1}, V_{k_2}, \cdots, V_{k_m}$ to V_j is globally identifiable. That is, the Wright's coefficient of each detour-path in S_IDP is globally identifiable.

Lemma 4. Given a DAG $\mathrm{G}=(\mathbf{V},\mathbf{E})$, an observed node V_i, and an unobserved node V_u in $O^{(k)}$, if only V_u becomes observed in $O^{(k+1)}$, there exist two cases:

1) each Wright's path between V_i and V_u passes at least one observed node other than V_i and V_u when none of the Wright's paths between V_i and V_u contains detour-paths;
2) each Wright's path between V_i and V_u passes at least one observed node other than V_i and V_u, and the Wright's coefficient of each detour-path between V_i and V_u is globally identifiable in $O^{(k)}$ when certain Wright's paths between V_i and V_u contain detour-paths.

Then the identifiability equation $IE(V_i, V_u)$ is redundant if and only if one of the above conditions holds.

Proof. We first prove the sufficient condition for the first case. Assume that V_i is an ancestor node of V_u, and there exist m Wright's paths between V_i and V_u, and each path passes an observed node that is not a collider node of the detour-paths, denoted by $V_1, V_2, \cdots, V_m$ (note that such nodes are in $O^{(k)}$ and thus also in $O^{(k+1)}$), respectively. Let S_WP_{pq} denote the sum of all the Wright's path coefficients between V_p and V_q, i.e., $S\_WP_{pq}=\sum_r WP_r$; then we can get C_{m+1}^2 identifiability equations from $O^{(k)}$ because one identifiability equation can be generated for each pair of *d*-connected observed nodes [3, 4]. There are C_m^2 identifiability equations between any two nodes of $V_1, V_2, \cdots, V_m$ and m identifiability equations between V_i and each of $V_1, V_2, \cdots, V_m$. Although there exist some Wright's paths among $V_1, V_2, \cdots, V_m$ that do not pass V_i, here we ignore this case and focus only on the case that all the Wright's

paths among $V_1, V_2, \cdots, V_m$ pass V_i. Now we can get the following identifiability equations,

$$IE(V_i, V_1): Cov(V_i, V_1) = S\_WP_{1i},\ \ IE(V_i, V_2):\ Cov(V_i, V_2) = S\_WP_{2i},\ \ \cdots,$$

$$IE(V_i, V_m):\ Cov(V_i, V_m) = S\_WP_{mi}.$$

Consider an identifiability equation $IE(V_p, V_q)$ between two nodes $V_p, V_q \in \{V_1, V_2, \cdots, V_m\}$, we get

$$IE(V_p, V_q):\ Cov(V_p, V_q) = S\_WP_{pq} = S\_WP_{ip} \cdot S\_WP_{iq} = Cov(V_i, V_p) \cdot Cov(V_i, V_q).$$

Since $IE(V_p, V_q)$ does not contain any unknown parameters, it is redundant. This means that these C_m^2 identifiability equations among $V_1, V_2, \cdots, V_m$ can be ignored given the existing identifiability equations in $O^{(k)}$. When V_u becomes observed in $O^{(k+1)}$, the following $k+1$ identifiability equations are newly added

$$IE(V_u, V_1):\ Cov(V_u, V_1) = S\_WP_{1u},\qquad IE(V_u, V_2):\ Cov(V_u, V_2) = S\_WP_{2u}, \cdots,$$

$$IE(V_u, V_m):\ Cov(V_u, V_m) = S\_WP_{mu},\qquad IE(V_u, V_i):\ Cov(V_u, V_i) = S\_WP_{iu}.$$

Consider an observed node $V_l \in \{V_1, V_2, \cdots, V_m\}$. If some Wright's paths between V_i and V_u pass V_l, then these paths can be divided into two parts: one between V_i and V_l, and the other between V_l and V_u. The Wright's path coefficient sum of the first part and the second part are just S_WP_{li} and S_WP_{lu}, respectively. Then

$$IE(V_j, V_l):\ Cov(V_j, V_l) = S\_WP_{lu} = S\_WP_{lu} + \sum_{p=1,\, p\neq l}^{m} S\_WP_{il} \cdot S\_WP_{ip} \cdot S\_WP_{pu}.$$

Substitute $IE(V_i, V_l):\ Cov(V_i, V_l) = S\_WP_{il}$ and $IE(V_i, V_p):\ Cov(V_i, V_p) = S\_WP_{ip}$ into the equation above, we get

$$IE(V_u, V_l): Cov(V_u, V) = S\_WP_{lu} = S\_WP_{lu} + \sum_{p=1,\, p\neq l}^{m} Cov(V_i, V_l) \cdot Cov(V_i, V_p) \cdot S\_WP_{pu}.$$

There are m equations between V_u and V_l, and m unknown terms $S\_WP_{lu}\left(l=1,2,\cdots,m\right)$ in all the identifiability equations $IE\left(V_u,V_l\right)$. Furthermore, all the identifiability equations $IE\left(V_u,V_l\right)$ are linearly independent. One can tell that all the unknown terms S_WP_{lu} can be uniquely determined from $IE\left(V_u,V_l\right)$ $\left(l=1,2,\cdots,m\right)$.

Now we consider the identifiability equation $IE\left(V_i,V_u\right)$,

$$IE\left(V_i,V_u\right):\ Cov\left(V_i,V_u\right)=S\_WP_{iu}=\sum_{l=1}^{m}S\_WP_{il}\cdot S\_WP_{lu}=\sum_{l=1}^{m}Cov\left(V_i,V_l\right)\cdot S\_WP_{lu}.$$

Since S_WP_{lu} can be uniquely determined by equations $IE\left(V_u,V_l\right)\left(l=1,2,\cdots,m\right)$, $IE\left(V_i,V_u\right)$ contains no unknown parameters and it can be expressed as a linear combination of other identifiability equations. Therefore, $IE\left(V_i,V_u\right)$ is redundant. Thus, the sufficient condition holds for the first case.

Next we prove the necessary condition for the first case by contradiction. Without loss of generality, assume that V_i is an ancestor node of V_u and $IE\left(V_i,V_u\right)$ is redundant, but there exists a Wright's path P_{iu} between V_i and V_u passes none of the observed nodes, while the other m Wright's paths pass m observed nodes $V_1,V_2,\cdots,V_m$, respectively. As before, we consider the case that all the Wright's paths among $V_1,V_2,\cdots,V_m$ pass V_i and ignore the case that some Wright's paths among $V_1,V_2,\cdots,V_m$ do not pass V_i. Then we can get m identifiability equations between V_i and each of $V_1,V_2,\cdots,V_m$ from $O^{(k)}$, and ignore the C_m^2 identifiability equations among $V_1,V_2,\cdots,V_m$. Moreover, we can get $m+1$ new identifiability equations when V_j becomes observed in $O^{(k+1)}$. Let WP_{iu} denote the Wright's path coefficient of path P_{iu}, then we have

$$IE\left(V_i,V_u\right):\ Cov\left(V_i,V_u\right)=\sum_{l=1}^{m}S\_WP_{il}\cdot S\_WP_{lu}+WP_{iu}=\sum_{l=1}^{m}Cov\left(V_i,V_l\right)\cdot S\_WP_{lu}+WP_{iu}.$$

Because V_u is unobserved in $O^{(k)}$, all the identifiability equations from $O^{(k)}$ do not contain the term WP_{iu}. Similarly, all the newly added identifiability equations except for $IE\left(V_i,V_u\right)$ from $O^{(k+1)}$ do not contain WP_{iu} because P_{iu} does not pass any observed node. This is, $IE\left(V_i,V_u\right)$ contains the term WP_{iu} that does not appear in any other identifiability equations. Therefore, the identifiability equation $IE\left(V_i,V_u\right)$ cannot be expressed as a linear combination of other identifiability equations, and thus $IE\left(V_i,V_u\right)$ is not redundant, which contradicts to the assumption of $IE\left(V_i,V_u\right)$ being redundant. Therefore, the necessary condition holds for the first case that none of the Wright's paths between V_i and V_u contains detour-paths.

Then we prove the sufficient condition for the second case. Assume that V_i is an ancestor node of V_u, and there exist m Wright's paths that contain detour-paths and the Wright's coefficient of each detour-path is globally identifiable in $O^{(k)}$, and n Wright's paths that do not contain detour-paths and pass at least one observed node other than V_i and V_u. Let $V_{k_p}\left(q=1,2,\cdots,m\right)$ denote the collider nodes of m Wright's paths with detour-paths and let $V_{k_q'}\left(p=1,2,\cdots,n\right)$ denote the observed nodes of n Wright's paths without detour-paths, respectively. Correspondingly, V_i is the upstream node and V_u is the downstream node of all the detour-paths. Similar to the first case, we can get $\left(m+n\right)$ non-redundant identifiability equations between V_i and all the nodes in $V_{k_1},V_{k_2},\cdots,V_{k_m}$ and $V_{k_1'},V_{k_2'},\cdots,V_{k_n'}$ from $O^{(k)}$, and $\left(m+n+1\right)$ new identifiability equations when V_u becomes observed in $O^{(k+1)}$, and we have

$$IE\left(V_i,V_u\right):\ Cov\left(V_i,V_u\right)=\sum_{l=1}^{n}Cov\left(V_i,V_{k_l^{'}}\right)\cdot S\_WP_{k_l^{'}u}+\sum_{r=1}^{m}Cov\left(V_i,V_{k_r}\right)\cdot S\_DWP_{k_r u},$$

where $S\_WP_{k_l^{'}u}$ can be uniquely determined by $IE\left(V_u,V_{k_l^{'}}\right)\left(l=1,2,\cdots,n\right)$, and $S\_DWP_{k_r u}$ denotes the Wright's path coefficient sum of all the detour-paths from V_{k_r} to V_u. Because each detour-path is globally identifiable in $O^{(k)}$ (i.e., $S\_DWP_{k_r u}$ is globally identifiable), one can tell that $IE\left(V_i,V_u\right)$ does not contain any unknown parameters (i.e., $IE\left(V_i,V_u\right)$ can be expressed as a linear combination of other identifiability equations), and thus $IE\left(V_i,V_u\right)$ is redundant. Therefore, the sufficient condition holds for the second case.

Finally, we prove the necessary condition for the second case by contradiction. We assume that there exists a Wright's path P_{iu} between V_i and V_u that passes no observed nodes or there exists one detour-path, the Wright's coefficient of which is unidentifiable (note that there are only two cases: globally identifiable and unidentifiable for a detour-path), but $IE\left(V_i,V_u\right)$ is redundant. Same as the first case, if there exists a Wright's path P_{iu} between V_i and V_u passing no observed nodes, then $IE\left(V_i,V_u\right)$ is not redundant. This contradicts to the assumption of $IE\left(V_i,V_u\right)$ being redundant. Now consider the case that there exist one detour-path, the Wright's coefficient of which is unidentifiable. Similar to the proof of the sufficient condition, we can get

$$IE\left(V_i,V_u\right):\ Cov\left(V_i,V_u\right)=\sum_{l=1}^{n}Cov\left(V_i,V_{k_l^{'}}\right)\cdot S\_WP_{k_l^{'}u}+\sum_{r=1}^{m}Cov\left(V_i,V_{k_r}\right)\cdot S\_WP_{k_r u}.$$

If there exists one detour-path with an unidentifiable Wright's coefficient (i.e., there exists one unidentifiable $S\_DWP_{k_r u}$), this means that the identifiability equation

$IE(V_i, V_u)$ contains one term that cannot be expressed as a linear combination of other identifiability equations, and thus $IE(V_i, V_u)$ is not redundant, which contradicts to the assumption of $IE(V_i, V_u)$ being redundant. Therefore, the necessary condition holds for the second case. In summary, the lemma holds. ■

Lemma 5. Given a DAG $\mathrm{G} = (\mathbf{V}, \mathbf{E})$, two *d*-connected observed nodes V_i and V_j, and an unobserved node V_u in $O^{(k)}$, if V_u is on a Wright's path between V_i and V_j and only V_u becomes observed in $O^{(k+1)}$, there exist two cases:

1) each Wright's path between V_i and V_j passes at least one observed node other than V_i and V_j when none of the Wright's paths between V_i and V_j contains detour-paths;
2) each Wright's path between V_i and V_j passes at least one observed node other than V_i and V_j, and the Wright's coefficient of each detour-path between V_i and V_j is globally identifiable in $O^{(k)}$ when certain Wright's paths between V_i and V_j contain detour-paths.

Then one of the two identifiability equations $IE(V_i, V_u)$ and $IE(V_j, V_u)$ is redundant if and only if one of the above conditions holds.

Proof. We can get the identifiability equation $IE(V_i, V_j)$ from $O^{(k)}$. After V_u becomes observed in $O^{(k+1)}$, two new identifiability equations $IE(V_i, V_u)$ and $IE(V_j, V_u)$ can be obtained. Similar to Lemma 4, $IE(V_i, V_j)$ can be expressed as a linear combination of other identifiability equations. This means that one of $IE(V_i, V_u)$ and $IE(V_j, V_u)$ can also be expressed as a linear combination of other identifiability equations, i.e., one of the identifiability equations $IE(V_i, V_u)$, $IE(V_j, V_u)$ is

redundant. The proof details are similar to those of Lemma 4 and thus skipped. ■

Theorem 1. Given a DAG $\mathrm{G}=(\mathbf{V},\mathbf{E})$ and an unobserved node V_i in an observation strategy O, let G' denote the sub-graph after the edge-removal operation. Then the identifiability gain is $g(V_i,O)=N_w-N_r$, where N_w denotes the total number of the observed nodes that are connected with V_i via any Wright's path in graph G', and N_r denotes the number of redundant identifiability equations in graph G'.

Proof. Because G is a DAG, all the nodes of G except for V_i can be classified into three sets: anc_i, des_i and rel_i. After V_i becomes observed, the number of newly-added identifiability equations is the sum of the numbers of observed nodes that are *d*-connected with V_i in anc_i, des_i or rel_i [3, 4]. Also, let S_WP_{pq} denote the sum of all the Wright's path coefficients between node V_p and node V_q.

First, for an observed node V_j in anc_i, the Wright's paths between V_i and V_j are just the directed paths from V_j to V_i, and the corresponding identifiability equation is $IE(V_i,V_j)$: $Cov(V_i,V_j)=S\_WP_{ij}$ if V_i becomes observed. If $IE(V_i,V_j)$ is redundant in the case that none of the Wright's paths between V_i and V_j contains detour-paths, each path P_l from V_j to V_i will pass at least one observed node $V_k\,(k\neq i,j)$ according to Lemma 4. After removing all the incoming edges to the observed nodes that are not collider nodes of the detour-paths in S_AV_i, the intermediate observed node V_k on path P_l loses its incoming edges such that V_i will be disconnected with V_j. If $IE(V_i,V_j)$ is non-redundant in the case that none of the Wright's paths between V_i and V_j contains detour-paths, then there exists at least

one path from V_j to V_i that does not pass any observed node that is not a collider of the detour-paths or has a length of 1. Such paths will not be affected by removing the incoming edges to the observed nodes. Thus, in graph G', node V_i is connected with V_j in S_AV_i if $IE\left(V_i,V_j\right)$ is not redundant in the case that none of the Wright's paths between V_i and V_j contains detour-paths, but disconnected with V_j in S_AV_i if $IE\left(V_i,V_j\right)$ is redundant when none of the Wright's paths between V_i and V_j contains detour-paths.

Second, for an observed node V_j in des_i, the Wright's paths between V_i and V_j are just the paths from V_i to V_j. The identifiability equation $IE\left(V_i,V_j\right)$ is not redundant in the following three cases: 1) at least one Wright's path has a length of 1; 2) at least one Wright's path does not pass any observed nodes; 3) at least one Wright's path contains one detour-path with its Wright's coefficient being unidentifiable. Similar to the previous case, after removing all the outgoing edges from the observed nodes that are not the colliders of the detour-paths with unidentifiable Wright's coefficients in des_i, in graph G', node V_i is still connected with V_j in des_i if $IE\left(V_i,V_j\right)$ is not redundant and disconnected with V_j in des_i if $IE\left(V_i,V_j\right)$ is redundant.

Finally, for an observed node V_j in rel_i, the identifiability equation is $IE\left(V_i,V_j\right)$: $Cov\left(V_i,V_j\right)=\sum_{V_k\in S\_BV_i} WP_{ik}\cdot WP_{kj}$. In other words, each Wright's path P_l between V_i and V_j consists of two segments: one from V_k to V_i and the other from V_k to V_j. If $IE\left(V_i,V_j\right)$ is redundant, then each Wright's path contains at least one observed node V_l according to Lemma 4. This observed node V_l may be in one of the three sets: 1) $anc_i-bound_i$; 2) $bound_i$; 3) rel_i. When $V_l\in\left\{anc_i-bound_i\right\}$,

after removing all the incoming edges to the observed nodes that are not the colliders of detour-paths in anc_i, the path from V_k to V_i is broken, and correspondingly the original Wright's path P_l does not exist in graph G'. This is, nodes V_i and V_j are disconnected in G' if $IE(V_i,V_j)$ is redundant in the case that none of the Wright's paths between V_i and V_j contains detour-paths and $V_l \in \{anc_i - bound_i\}$. When $V_l \in bound_i$, after removing all the outgoing edges from the observed nodes in $bound_i$ to nodes in rel_i, the path from V_k to V_j is broken, and correspondingly the original Wright's path P_l does not exist in graph G'. This is, nodes V_i and V_j are disconnected in graph G' if $IE(V_i,V_j)$ is redundant and $V_l \in bound_i$. When $V_l \in rel_i$, after removing all the outgoing edges from the observed nodes that are not the colliders of the detour-paths with unidentifiable Wright's coefficients in rel_i, the path from V_k to V_j is broken, and correspondingly the original Wright's path P_l does not exist in graph G'. One can tell that nodes V_i and V_j are disconnected in graph G' if $IE(V_i,V_j)$ is redundant and $V_l \in rel_i$, and connected in graph G' if $IE(V_i,V_j)$ is not redundant.

In summary, after the edge-removal operation, for each observed node in G' that connects with V_i, and one identifiability equation can be generated. Among these equations, there still exist some redundant identifiability equations, because there are two cases that are not dealt with by the edge-removal operation: 1) One edge-removal operation is to remove all the incoming edges to the observed nodes that are not the colliders of detour-paths in anc_i, and this edge-removal process ignores the case that the intermediate observed nodes are the colliders of detour-paths in anc_i; 2) all the

edge-removal operations do not consider the case that V_i is a collider of detour-paths. These two cases still exist in the sub-graph G'. Let N_w denote the total number of the observed nodes that are connected with V_i via any Wright's path in graph G', and let N_r denote the number of redundant identifiability equations in graph G'. Therefore, by definition, the identifiability gain is $g(V_i, O) = N_w - N_r$. The theorem holds. ■

Lemma 6. For a given DAG $\mathrm{G} = (\mathbf{V}, \mathbf{E})$, the following nodes must be observed to assure that all the parameters of the corresponding SEM are at least locally identifiable

1) The nodes with an out-degree 0;
2) The nodes with an out-degree 1;
3) The nodes with an in-degree 0 and an out-degree less than 3.

Proof. 1) Consider an unobserved node V_i with an out-degree 0 in G, as shown in Fig. S-2(a). According to the Wright's path coefficient method [1, 2], the parameters associated with all the incoming edges to V_i are not contained in any identifiability equation since V_i is a collider; thus, all the incoming edge parameters of V_i are unidentifiable. That is, the nodes with an out-degree 0 must be observed.

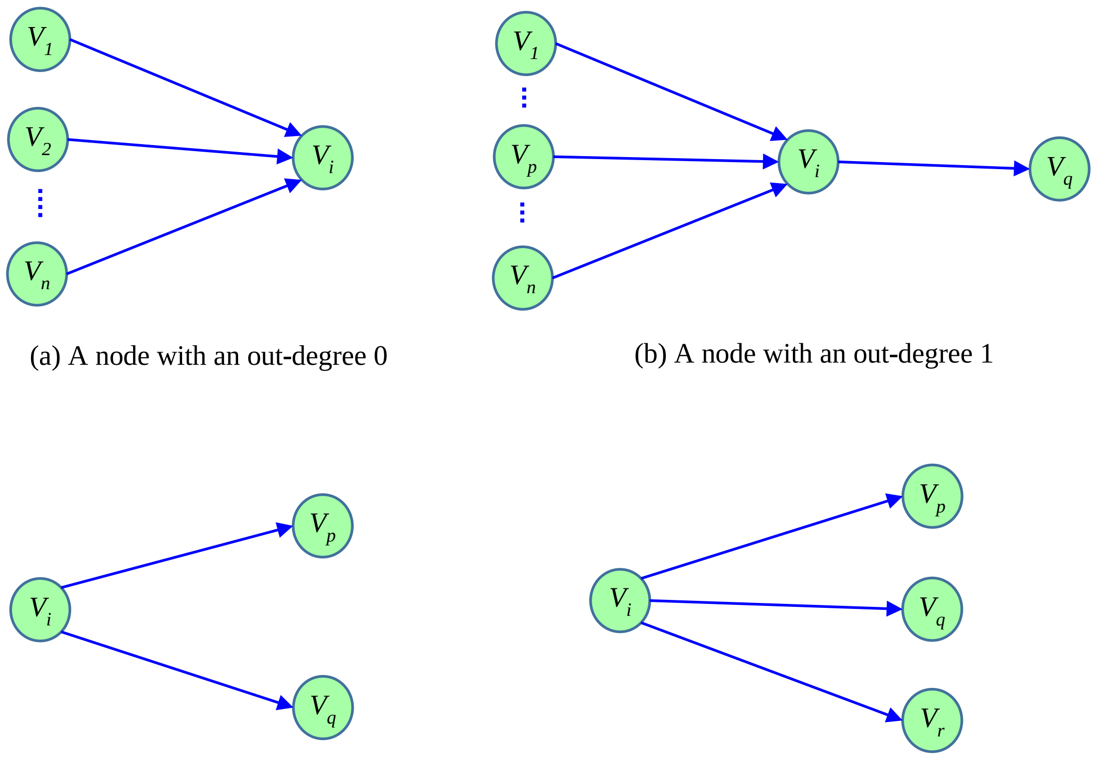

(a) A node with an out-degree 0

(b) A node with an out-degree 1

(c) A node with an out-degree 2

(d) A node with an out-degree 3

Figure S-2. Illustration of the must-be-observed nodes.

2) Consider an unobserved node V_i with an out-degree 1 in G and an in-degree n $(n = 0,1,2,...)$. When $n = 0$, the parameter c_i associated with the outgoing edge from V_i is not contained in any identifiability equations. Then c_i is unidentifiable, and V_i must be observed when V_i has an out-degree 1 and an in-degree 0. When $n > 0$, we first consider the case in Fig. S-2(b), where there exist no edges between the in-neighbor nodes of V_i. When all the neighbors of V_i are observed, we can get one identifiability equation $IE(V_p, V_q)$ for each in-neighbor node V_p and the out-neighbor node V_q. Because there are n in-neighbor nodes, we can get n identifiability equations. However, there are $n+1$ unknown parameters in these identifiability equations (i.e., n incoming edge parameters and one outgoing edge parameter). Thus, the $n+1$ unknown parameters are unidentifiable.

Even if there exist some edges between the in-neighbor nodes of V_i, the newly generated identifiability equations among the in-neighbor nodes will not contain any of the unknown parameters associated with the incoming or outgoing edges of V_i because V_i is a collider with respect to the in-neighbor nodes. Therefore, the nodes with an out-degree 1 must be observed.

3) There are two cases to consider here: the nodes with an in-degree 0 and an out-degree 1, and the nodes with an in-degree 0 and an out-degree 2. The first case has been discussed in Fig. S-2(b), so we focus on the second case. As shown in Fig. S-2(c), where there are no edges connecting the two out-neighbor nodes V_p and V_q. When the two out-neighbor nodes are observed, we can get only one identifiability equation $IE\left(V_p, V_q\right)$, but this identifiability equation contains two unknown parameters (i.e., the parameters associated with the two outgoing edges of V_i). Therefore, the two outgoing edge parameters are unidentifiable. Second, when there is one edge between two out-neighbor nodes V_p and V_q, still only one identifiability equation $IE\left(V_p, V_q\right)$ can be generated, but now it contains three unknown parameters (i.e., two outgoing edge parameters and one edge parameter between V_p and V_q). So the two outgoing edge parameters are still unidentifiable.

When there are more descendent nodes of V_i and more edges among the nodes, more identifiability equations will be obtained. However, these identifiability equations cannot help to verify the identifiability of the outgoing edge parameters of V_i because the two outgoing edge parameters always appear together in forms of a product in any identifiability equation. Therefore, the nodes with an in-degree 0 and an out-degree 1

or 2 must be observed.

Finally, consider an unobserved node with an in-degree 0 and an out-degree 3. We start with the case shown in Fig. S-2(d), where there are no edges between the three out-neighbor nodes V_p , V_q and V_r. When all the out-neighbor nodes are observed, we can get three identifiability equations: $IE\left(V_p,V_q\right)$, $IE\left(V_p,V_r\right)$ and $IE\left(V_q,V_r\right)$. These three identifiability equations contains three unknown parameters (i.e., the three outgoing edge parameters of V_i), and these equations are non-redundant. Therefore, the three outgoing-edge parameters are at least locally identifiable when all the out-neighbor nodes are observed. For an unobserved node with an in-degree 0 and an out-degree greater than 3, we can reach the same conclusion. Therefore, an unobserved node with an in-degree 0 and an out-degree equal to or greater than 3 is not required to be observed.

In summary, the lemma holds. ■

References

1. Wright S. The method of path coefficients. The Annals of Mathematical Statistics. 1934;5(3):161-215.

2. Wright S. Path coefficients and path regressions: alternative or complementary concepts? Biometrics. 1960;16. doi: 10.2307/2527551.

3. Pearl J. Causality: models, reasoning, and inference (2nd Edition). Cambridge: Cambridge University Press; 2009.

4. Pearl J. The causal foundations of structural equation modeling. DTIC Document, 2012.
